# Paroxysmal and Non-Paroxysmal Atrial Fibrillation in Middle Eastern Patients: Clinical Features and the Use of Medications. Analysis of the Jordan Atrial Fibrillation (JoFib) Study

**DOI:** 10.3390/ijerph19106173

**Published:** 2022-05-19

**Authors:** Hanna Al-Makhamreh, Nasr Alrabadi, Lubna Haikal, Mohammad Krishan, Noor Al-Badaineh, Osama Odeh, Tawfiq Barqawi, Mohammed Nawaiseh, Ala Shaban, Basil Abdin, Lama Khamies, Ayman Hammoudeh

**Affiliations:** 1 Department of Cardiology, School of Medicine, University of Jordan, Amman 11972, Jordan; 2 Department of Pharmacology, Faculty of Medicine, Jordan University of Science and Technology, Irbid 22110, Jordan; lkalburie20@med.just.edu.jo; 3 School of Medicine, University of Jordan, Amman 11972, Jordan; lubna.haikal96@gmail.com (L.H.); mkraishan97@gmail.com (M.K.); noor.badaineh@hotmail.com (N.A.-B.); osamaodeh810@gmail.com (O.O.); t.barqawi.97@gmail.com (T.B.); basel.abdeen97@gmail.com (B.A.); 4 King Hussein Medical Center, Royal Medical Services, Amman 11855, Jordan; mohammednawaiseh.md@gmail.com; 5 King Hussein Cancer Center, Amman 11941, Jordan; a.shabban@hotmail.com; 6 Department of Cardiology, Istishari Hospital, Amman 11184, Jordan; hammoudeh_ayman@yahoo.com

**Keywords:** atrial fibrillation (AF), paroxysmal, non-paroxysmal, anticoagulants, arrhythmias

## Abstract

(1) Background: Atrial fibrillation (AF) is the most common arrhythmia causing an increased risk of mortality and morbidity. It is classified into paroxysmal and non-paroxysmal AF depending on the duration and frequency of the episodes. (2) Aims: Our goal was to investigate and compare the clinical profiles, risk of co-morbidities, the use of oral anticoagulation, and outcomes of patients with paroxysmal and non-paroxysmal AF in inpatient and outpatient settings. (3) Methods: Data were extracted from 28 different hospitals and centers in Jordan with a total of 2160 patients enrolled in the study using an observational non-interventional study model. The clinical features and the use of oral anticoagulants were compared in patients with paroxysmal and non-paroxysmal AF. (4) Results: Paroxysmal AF was documented in 35.6% (769) of the patients and non-paroxysmal types in 63.9% (1380); in addition, the type of AF was unknown in 11 (0.5%) patients. Our results showed that non-paroxysmal AF patients tend to be older with more co-morbidities and higher CHA2DS2-VASC and HAS-BLED scores. They also have higher rates of hypertension and diabetes. Anticoagulant, antiarrhythmic, and diuretic agents, overall, were used more in non-paroxysmal AF than paroxysmal AF. Hospital admissions were also more frequent in non-paroxysmal AF due to various factors, some of which are heart failure, bleeding risk, and COPD. (5) Conclusions: Non-paroxysmal AF is more common among Jordanian AF patients. The prevalence of comorbidities and the use of different types of therapies, especially anticoagulants, were higher in these patients.

## 1. Introduction

Atrial fibrillation (AF) is the most common arrhythmia affecting the heart with an estimated lifetime risk of about 22–26% [1] and a prevalence of about 1% in the general population [2] and 9% in adults aged 80 years or older [3]. It is characterized by rapid and unsynchronized atrial excitation which leads to impaired atrial function [4]. Hypertension, diabetes mellitus, smoking, heart failure, and valvular heart disease are some of the major risk factors for the development of atrial fibrillation [5]. AF is also associated with significant morbidity and mortality, including stroke, systemic embolization, heart failure, or even sudden cardiac death. Atrial fibrillation can be categorized as paroxysmal (reverts to sinus rhythm within 7 days), persistent (lasts more than 7 days), or long-standing persistent (lasts for more than 12 months). On the other hand, permanent AF is when a decision is made not to pursue further action to restore or maintain sinus rhythm [6].

The annual rate of stroke in patients with paroxysmal AF may be similar to permanent AF at 3.3% [7], suggesting that paroxysmal AF can also lead to serious sequela similar to other types of AF. However, due to its paroxysmal character, it is often underdiagnosed and undertreated [8]. In agreement, a UK study showed that patients with paroxysmal AF, who were eligible for anticoagulation, were 20% less likely to be prescribed medication [9]. This is of great value to know given that one of the main aims of treatment in AF patients is to reduce the risk of complications such as thromboembolis besides trying to prevent paroxysms and maintain sinus rhythm [10]. On the other hand, paroxysmal AF was found to progress to permanent AF in 77% of patients over 14 years at the beginning of the disease. Some risk factors such as age, left atrial dilatation, valvular disease, and myocardial infarction were recognized as independent risk factors for the progression to persistent AF [11].

There is a scarcity of comprehensive studies in the Middle East that evaluate the clinical characteristics of patients with paroxysmal and non-paroxysmal AF. Moreover, the sample size in these studies is small and their aims were not to focus on understanding the differences in the clinical profiles of paroxysmal and non-paroxysmal AF. Moreover, with the fast pace of urbanization in the Middle East in general, and in Jordan in particular, cardiovascular risk factors are increasing in prevalence along with the increase in life expectancy. These factors are contributing to a prelude to increases in AF incidence, prevalence, and possible changes in clinical profiles, thus, studies addressing the profiles of different types of AF are warranted. In the current study, we focused on comparing the clinical profiles and the use of oral anticoagulants (OACs) in a large and representative number of patients with paroxysmal and non-paroxysmal atrial fibrillation.

## 2. Materials and Methods

### 2.1. Study Design and Setting

This is a multi-center prospective observational non-interventional study that evaluated patients with AF in an in-patient and out-patient setting. Patients with AF from 28 Jordanian major hospitals and outpatient clinics were recruited for the aim of this study over one year. The data collection was extended from May 2019 to January 2021.

The inclusion criteria included: aged 18 years or above, admission with AF or clinical visit for AF, and signing an informed consent form to participate in the study. The exclusion criteria included: aged less than 18, and refusal to sign the consent form.

The type of AF was categorized into two groups. The first group included patients with paroxysmal AF and the second group non-paroxysmal AF, included persistent, long-standing, and permanent AF. More specifically, the categorization was based on the AHA/ACC recent guidelines on AF. Paroxysmal AF is defined as recurrent, ECG-documented, episodes of AF or a single ECG-documented episode of AF plus a patient history, indicating further episodes of AF. All episodes of AF are self-terminating within seven days of onset. Persistent AF, on the other hand, is defined as AF for which cardioversion was performed or planned, or AF lasting more than seven days that did not fulfill the criteria for permanent AF, while longstanding persistent is defined as an episode of atrial fibrillation known to have lasted longer than 12 months. Finally, permanent AF is defined as an acceptance of AF as the chronic rhythm, whereby the provider and patient have agreed to abandon further efforts to restore normal heart rhythm [6]. The CHA2DS2-VASc score was categorized into a high-risk group including males with a score of ≥2 points and females with a score of ≥3 points, in addition to a low/intermediate-risk group including males with a score of <2 for and females with a score of <3. Similarly, the HAS-BLED score was categorized into high-risk group (≥3) and low/moderate risk group (<3). All study protocols and patients’ categorization were approved by the participated clinicians before the start of the study and unified in all the 28 hospitals.

### 2.2. Statistical Analysis

Statistical analysis was conducted using SPSS v.25. Data were described using frequency and percentage for categorical variables and mean (±SD) for continuous variables. The mean values of continuous variables were compared between paroxysmal and non-paroxysmal AF using an independent samples t-test. The Chi-square test was used to study the differences among categorical variables between the two groups and to compare the frequency of AF symptoms between males and females among patients with paroxysmal and non-paroxysmal AF. Predictor variables with *p* values of less than 0.05 in the univariate analysis were entered into a multivariable binary logistic regression analysis to determine associations between the dependent and independent variables. Odds Ratios (OR) and 95% confidence intervals (CI) were calculated. Variance inflation factor (VIF) of more than 10 was set to exclude multi-colinear variables. To avoid any incorrect adjustments, variables used in the CHA2DS2-VASc score calculation were not included in the model. Reasons for admission, admission outcome, and cause of hospital death among AF patients who were admitted to the hospital (Inpatients) were compared between paroxysmal and non-paroxysmal AF. All statistical tests were two-sided, with *p* values <0.05 considered to be statistically significant. All underlying assumptions were met unless otherwise indicated.

## 3. Results

This study included 2160 AF patients, of whom 996 (46.2%) were males and 1164 (53.8%) were females. The mean age of the whole study sample was 67.8 (±13.0) years. Those who had paroxysmal AF numbered 769 (35.6%) and those who had non-paroxysmal types 1380 (63.9%). The type of AF was unknown in 11 patients (0.5%). Patients with non-paroxysmal AF included 326 (15.1%) patients with persistent AF, 435 (20.1%) patients with long-standing AF, and 619 (28.7%) patients with permanent AF. Tests to see if the data met the assumption of collinearity indicated that multicollinearity was not a concern between continuous variables (age and left atrial size; Tolerance = 0.957, VIF = 1.045).

Univariate analysis using Chi-square test and independent samples t-test (Table 1) revealed that the following variables were significantly higher among patients with non-paroxysmal AF compared to patients with paroxysmal AF: stroke and systemic embolization (18.9% vs. 15.5%, *p* = 0.045), age (70.2 ± 11.2 years vs. 63.7 ± 14.7 years, *p* < 0.001), hypertension (76.7% vs. 71.0%, *p* = 0.004), diabetes mellitus (46.7% vs. 39.1%, *p* = 0.001), high risk CHA2DS2-VASc score (86.4% vs. 67.5%, *p* < 0.001), high risk HAS-BLED score (23.2% vs. 13.8%, *p* < 0.001), left ventricle hypertrophy (41.5% vs. 36.0%, *p* = 0.017), severe left ventricle ejection fraction reduction ([LVEF], <30%, 7.0% vs. 2.0%, *p* < 0.001), mild-moderate LVEF reduction (30–50%, 24.1% vs. 11.9%, *p* < 0.001), left atrial size (4.5 ± 0.7 vs. 4.0 ± 0.7, *p* < 0.001), valvular heart disease (10.6% vs. 4.8%, *p* < 0.001), asymptomatic AF (35.4% vs. 25.5%, *p* < 0.001), shortness of breath (35.9% vs. 30.7%, *p* = 0.015), pulmonary hypertension (30.6% vs. 17.3%, *p* < 0.001), chronic kidney disease (10.2% vs. 7.3%, *p* = 0.024), and heart failure (31.1% vs. 11.8%, *p* < 0.001).

Moreover, some other, but fewer, variables were significantly higher among patients with paroxysmal AF compared to patients with non-paroxysmal AF, as follows: current smoking (17.1% vs. 11.4%, *p* < 0.001), first AF episode (49.3% vs. 16.4%, *p* < 0.001), and palpitations (55.4% vs. 36.7%, *p* < 0.001).

When the frequency of AF symptoms was compared between males and females among patients with paroxysmal and non-paroxysmal AF, we found that there was no statistically significant difference in AF symptoms among paroxysmal AF patients between the two genders. However, we found that palpitations (42.1% vs. 30.3%, *p* < 0.001), fatigue (28.6% vs. 17.2%, *p* < 0.001), dizziness (15.1% vs. 7.4%, *p* < 0.001) and shortness of breath (42.0% vs. 28.6%, *p* < 0.001) were more common in females compared to males among patients with non-paroxysmal AF, respectively.

Table 2 showcases the differences in frequency of various medications between the two groups. Warfarin, beta-blockers, non-dihydropyridine calcium channel blockers (CCB), digoxin, and diuretics were more commonly used in non-paroxysmal AF. Amiodarone, on the other hand, was more frequently used among paroxysmal AF patients.

As well, Beta-blockers were the most common medication consumed in both paroxysmal (82.0%) and non–paroxysmal (76.7%) AF. This was followed by direct oral anticoagulant (DOAC) for both groups. Those who had dual antiplatelets therapy among paroxysmal AF numbered 54 (7.0%) and among non-paroxysmal AF 87 (6.3%).

As shown in Table 3, the most common causes for admission among patients with paroxysmal AF were AF (41.9%) followed by non-cardiovascular causes (27.5%) and acute coronary syndrome (16.5%). On the other hand, the most common causes of admission among non-paroxysmal AF patients were non-cardiovascular (29.6%) causes followed by AF (22.9%) and heart failure (18.6%). Among those who were admitted to the hospital, 15 (6.4%) paroxysmal AF patients and 18 (4.6%) non-paroxysmal AF patients had died during their stay. More details about the causes of in-hospital deaths are presented in Table 3.

The multivariable binary logistic regression model was statistically significant with a likelihood ratio test χ2(17) = 475.01, *p* < 0.001 (Table 4). The regression model explained 32.3% (Nagelkerke R Square) of the variance. The following variables were significantly associated with the non-paroxysmal AF group: using digoxin (odds ratio (OR) = 2.39, *p* < 0.001, 95% confidence interval [CI] = 1.64 to 3.49), using warfarin (OR = 2.05, *p* < 0.001, 95% CI = 1.55 to 2.71), high risk CHA2DS2-VASc score (OR = 1.86, *p* < 0.001, 95% CI = 1.39 to 2.50), Left atrial size (OR = 1.61, *p* < 0.001, 95% CI = 1.36 to 1.92), CCB (OR = 1.60, *p* = 0.015, 95% CI = 1.09 to 2.33), shortness of breath (OR = 1.43, *p* = 0.011, 95% CI = 1.08 to 1.89), being asymptomatic (OR = 1.41, *p* = 0.050, 95% CI = 1.00 to 2.01), using beta blockers (OR = 1.41, *p* = 0.018, 95% CI = 1.06 to 1.89), and using diuretics (OR = 1.29, *p* = 0.038, 95% CI = 1.01 to 1.66). Moreover, presenting with first AF episode (OR = 0.23, *p* < 0.001, 95% CI = 0.18 to 0.30), having palpitations (OR = 0.65, *p* = 0.004, 95% CI = 0.49 to 0.87) and using amiodarone (OR = 0.66, *p* = 0.004, 95% CI = 0.50 to 0.87) were significantly associated with not being in the non-paroxysmal AF group.

## 4. Discussion

AF can be divided into paroxysmal (lasting 7 days or less, self-terminating) or non-paroxysmal (lasting more than 7 days, persistent, long-standing, or permanent) AF. This categorization can be useful in research; however, without definition of useful and clear clinical features of these types, it tends to mischaracterize the clinical reality. This study aims to recognize the differences between paroxysmal and non-paroxysmal AF in terms of clinical features, treatment modalities, and outcomes among Jordanian AF patients, thereafter building a useful and informative model to predict the disease prognosis, outcomes, and main themes for therapeutic approaches.

The study findings showed a clear shift of characteristics and outcomes between paroxysmal and non-paroxysmal AF patients, including risk factors, concomitant co-morbidities, thromboembolic and bleeding risk scores, and treatment modalities. These data and findings collected from the Jordanian population were found to be consistent with similar studies conducted in other populations globally [12]. For instance, our findings showed that non-paroxysmal AF patients tend to be older individuals with more co-morbidities and higher CHA2DS2-VASC and HAS-BLED scores than their paroxysmal counterparts.

Although previous studies have reached comparable conclusions to our study [13,14], conflicting results were also found when specific symptoms, concomitant diseases, and complications are considered. To elaborate on this point, we found that the most common symptom in both subtypes is palpitation; however, other studies showed dyspnea and fatigue as the dominant symptoms among non-paroxysmal subtypes [12,15].

On the other hand, we found an increase in the prevalence of dyspnea when progressing from paroxysmal to non-paroxysmal disease, a conclusion that was also reached by other studies. Asymptomatic patients in our cohort represented approximately one third or less in both groups (25.5% in paroxysmal and 35.4% in non-paroxysmal). In agreement with this, the EORP-AF Registry conducted on European populations showed close but higher results (36.9% in paroxysmal and 42.6% in non-paroxysmal according to EHRA score) [13], while another study concluded that paroxysmal AF patients tend to experience fewer symptoms than their non-paroxysmal counterparts [12].

Regarding the risk for stroke and systemic embolization, our results showed a higher risk in patients with non-paroxysmal AF than in those with paroxysmal AF. These results are also supported by other studies [14,16,17]. More specifically, with thromboembolism and bleeding risk assessment scores, our analysis yielded similar results to existing literature [13,18,19] where paroxysmal AF patients tend to be in the low-to-intermediate risk range of both ChA2DS2.-VASc and HAS-BLED scores more often than non-paroxysmal patients; as such, 67.5% and 86.4% of patients with paroxysmal and non-paroxysmal AF, respectively, qualified for oral anticoagulation.

Our results showed an increase in the frequency of hypertension and diabetes in non-paroxysmal AF when compared to paroxysmal AF. However, smoking (specifically current smoking) is more frequent in paroxysmal AF patients. This was in agreement with an international study conducted in 26 different countries [12], where it was found that hypertension was slightly more frequent among paroxysmal AF patients than in persistent or permanent AF patients. Diabetes was also found to be slightly more frequent in persistent and permanent AF when compared to paroxysmal AF. On the other hand, smoking was more frequent among persistent AF, followed by paroxysmal AF, then, lastly, permanent AF which may partially disagree with the findings of our study. Other comorbidities such as left ventricular hypertrophy, increased left atrial diameter, valvular disease, and heart failure were also more commonly seen in patients with non-paroxysmal AF, which agreed with other studies. However, we found conflicting results in the literature regarding the association of chronic kidney disease and pulmonary hypertension with non-paroxysmal AF [20,21,22,23].

The pharmacotherapy profile of patients in paroxysmal and non-paroxysmal AF patients was also reviewed in our study. In the oral anticoagulation category, warfarin was found to be used significantly highly in non-paroxysmal AF, a result supported by a study done in the U.S. [24] and by the ROCKET-AF trial [17], and this contradicted with the results obtained from the ARISTOTLE trial [14]. Our results could be explained by the fact that persistent and permanent AF carries a higher risk of thromboembolic events and stroke. Moreover, although the overall use of OACs and DOACs, in particular, is high in eligible patients with nonvalvular AF, we understand that some subgroups of patients with NVAF have paroxysmal AF and lower-than-average use of DOACs. Such subgroups include patients with low CHA2DS2-VASc scores and young patients.

In the category of antiarrhythmic medications, all agents listed in Table 2 had a higher percentage of usage by non-paroxysmal AF patients, except for amiodarone, which was prescribed more frequently in paroxysmal AF patients. Moreover, non-paroxysmal AF patients were treated with beta-blockers more often [15] and digoxin usage was significantly higher in non-paroxysmal AF patients [25]. These results could be explained by the utility of such agents in controlling ventricular rate in the long term by slowing AV-nodal conduction during AF [26]. Finally, our results also showed that the use of diuretics was significantly higher in non-paroxysmal AF patients, similar to a study done in Europe [13] and to the ROCKET-AF trial [17].

In this study, we also reviewed the in-hospital outcomes in paroxysmal and non-paroxysmal AF patients. We found that the AF itself is the most common cause of admission for paroxysmal AF patients. On the other hand, the non-paroxysmal patients were more often admitted for non-cardiovascular-related causes, with atrial fibrillation being the second most common cause for admission. This result might be explained by the higher prevalence of concomitant disease in non-paroxysmal AF patients. While the study of Lip et al. 2014 [27] corroborated these findings when it comes to paroxysmal AF, their data suggests that non-paroxysmal patients’ most common cause of admission was also atrial fibrillation, but it also points to a shift toward other causes of admission as the disease progresses from persistent to permanent, with heart failure being the second most common cause of admission in permanent AF patients (34.6% atrial fibrillation vs. 31.0% heart failure).

Our results also showed that heart failure tends to be more common as a cause of admission in non-paroxysmal patients when compared to patients in the paroxysmal subtype. A previously published study found that in non-paroxysmal AF there is an increased incidence of in-hospital admission due to heart failure [28]. The study of Taillandier et al. 2014 [29] showed an increased risk of admission in non-paroxysmal groups of patients because of heart failure when compared to the paroxysmal group.

Our results showed that hospital admissions due to bleeding risk and COPD are more frequently noticed in the non-paroxysmal subtype in comparison to paroxysmal AF. This is expected given the higher frequency of use of anticoagulants as previously mentioned. On the other hand, the results also showed similar risks between the studied groups in cerebrovascular accidents. However, an increased risk of systemic embolization in the non-paroxysmal group was noticed.

Moreover, the relation between the atrial fibrillation type and in-hospital mortality outcome in our study showed a higher incidence of in-hospital death in patients with paroxysmal AF, whereas, in terms of causes, sepsis was found to be the major cause of in-hospital death in our study, with 3.8% of paroxysmal AF patients admitted and 1.0% of admissions of non-paroxysmal AF patients.

Finally, it is important to note that our study showed that there were some gender disparities in the symptoms associated with AF and as expected they were more frequent among females [30,31]. However, this was found only in the non-paroxysmal AF group. More specifically, palpitation, fatigue, dizziness, and shortness of breath were more common among females only in non-paroxysmal AF patients. Many studies have discussed the gender disparities among AF patients [32,33]. However, there is a lack of studies exploring these differences in relation to the type of AF. Therefore, more studies are needed in this field.

### Limitations

While this study, just as any other observational study can hold a potential bias, we have taken great measures to reduce this. We recruited participants from 28 different hospitals and medical centers in Jordan that are representative of the health care system, involving public, private, and teaching sectors, thus improving the study’s generalizability. All of the participants are managed by cardiologists rather than general practitioners or any other type of specialists. These measurements were taken to reduce the chances of bias. However, we cannot ignore the possibility that variations between hospitals and clinicians can still exist.

## 5. Conclusions

Our study has provided a snapshot of data regarding risk factors and management of patients with paroxysmal and non-paroxysmal AF in Jordan. Around one-third of the patients had paroxysmal AF while two-thirds had non-paroxysmal AF. In general, the prevalence of comorbidities was higher in patients with non-paroxysmal AF, while symptoms were more prevalent in the paroxysmal AF group. The proportion of patients in both groups who qualified for anticoagulation was similar to the proportion that were on anticoagulation, suggesting adequate treatment.

## Figures and Tables

**Table 1:** Baseline features and characteristics of patients with paroxysmal and non-paroxysmal atrial fibrillation.

Variable	Paroxysmal (769)	Non–Paroxysmal (1380)	*p*-Value	Total (2160) *
Stroke and systemic embolization	119 (15.5%)	261 (18.9%)	0.045	380 (17.7%)
Male	366 (47.6%)	624 (45.2%)	0.288	989 (46.1%)
Female	403 (52.4%)	756 (54.8%)	0.288	1158 (53.9%)
Age	63.7 (14.7)	70.2 (11.2)	<0.001	
Body mass index (kg/m²)			0.084	
Normal (<25)	151 (21.0%)	303 (24.4%)		454 (23.2%)
Abnormal (≥25)	568 (79.0%)	938 (75.6%)		1506 (76.8%)
Hypertension	546 (71.0%)	1058 (76.7%)	0.004	1604 (74.6%)
Diabetes mellitus	301 (39.1%)	645 (46.7%)	0.001	946 (44.0%)
Current smoker	131 (17.1%)	158 (11.4%)	<0.001	289 (13.5%)
Dyslipidemia	349 (45.4%)	604 (43.8%)	0.488	953 (44.4%)
First atrial fibrillation episode	379 (49.3%)	227 (16.4%)	<0.001	606 (28.2%)
CHA2DS2-VASc score			<0.001	
Low and intermediate risk (<2 for males or <3 for females)	250 (32.5%)	188 (13.6%)		438 (20.4%)
High risk (≥2 for males or ≥3 for females)	519 (67.5%)	1190 (86.4%)		1709 (79.6%)
HAS-BLED score			<0.001	
Low and moderate risk (<3)	663 (86.2%)	1060 (76.8%)		1723 (80.2%)
High risk (≥3)	106 (13.8%)	320 (23.2%)		426 (19.8%)
Outpatient or inpatient			0.368	
Outpatient	533 (69.3%)	982 (71.2%)		1515 (70.5%)
Inpatient	236 (30.7%)	398 (28.8%)		634 (29.5%)
Left ventricle hypertrophy			0.017	
Present	247 (36.0%)	521 (41.5%)		768 (39.6%)
Absent	439 (64.0%)	733 (58.5%)		1172 (60.4%)
Left ventricle ejection fraction			<0.001	
Normal (≥50)	618 (86.2%)	905 (68.9%)		1523 (75.0%)
Mild-Moderate reduction (30–50)	85 (11.9%)	316 (24.1%)		401 (19.8%)
Severe reduction (<30)	14 (2.0%)	92 (7.0%)		106 (5.2%)
Left atrial size (centimetre)	4.0 (0.7)	4.5 (0.7)	<0.001	
Valvular vs. non-valvular atrial fibrillation			<0.001	
Valvular	37 (4.8%)	146 (10.6%)		183 (8.5%)
Non-valvular	732 (95.2%)	1234 (89.4%)		1966 (91.5%)
Symptoms				
Asymptomatic	196 (25.5%)	488 (35.4%)	<0.001	684 (31.8%)
Palpitations	426 (55.4%)	507 (36.7%)	<0.001	933 (43.4%)
Fatigue	156 (20.3%)	323 (23.4%)	0.096	479 (22.3%)
Dizziness	96 (12.5%)	160 (11.6%)	0.542	256 (11.9%)
Shortness of breath	236 (30.7%)	495 (35.9%)	0.015	731 (34.0%)
Syncope	22 (2.9%)	26 (1.9%)	0.142	48 (2.2%)
Chest pain	18 (2.3%)	17 (1.2%)	0.052	35 (1.6%)
Comorbid diseases				
Pulmonary Hypertension	133 (17.3%)	420 (30.6%)	<0.001	553 (25.9%)
Sleep apnea	33 (4.3%)	59 (4.3%)	0.986	92 (4.3%)
Lung disease (COPD or lung fibrosis)	29 (3.8%)	66 (4.8%)	0.274	95 (4.4%)
Thyroid disease	82 (10.7%)	148 (10.7%)	0.965	230 (10.7%)
CKD	56 (7.3%)	141 (10.2%)	0.024	197 (9.2%)
Active cancer	39 (5.1%)	78 (5.7%)	0.570	117 (5.4%)
Heart failure	91 (11.8%)	429 (31.1%)	<0.001	520 (24.2%)
Coronary artery disease	84 (10.9%)	151 (10.9%)	0.989	235 (10.9%)

CKD, Chronic kidney disease; COPD, Chronic obstructive pulmonary disease. * The type of AF was unknown in 11 patients (0.5%).

**Table 2:** Types of pharmacotherapies used in paroxysmal and non-paroxysmal AF patients.

Treatments/Drugs	Paroxysmal	Non-Paroxysmal	*p*-Value
Anticoagulant agents	506 (65.8%)	1188 (86.1%)	
Warfarin	141 (18.3%)	560 (40.6%)	<0.001
DOAC	365 (47.5%)	628 (45.5%)	0.383
Antiarrhythmic medications			
Beta blockers	590 (76.7%)	1132 (82.0%)	0.003
Amiodarone	207 (26.9%)	210 (15.2%)	<0.001
CCB (diltiazem or verapamil)	59 (7.7%)	173 (12.5%)	<0.001
Digoxin	68 (8.8%)	263 (19.1%)	<0.001
Antiplatelet agents			
Aspirin	299 (38.9%)	532 (38.6%)	0.880
Clopidogrel	119 (15.5%)	176 (12.8%)	0.079
Dual antiplatelets therapy	54 (7.0%)	87 (6.3%)	0.512
RAAS inhibitor (ACEi/ARB/Sacubitril/valsartan)	282 (36.7%)	549 (39.8%)	0.156
Statins	297 (38.6%)	513 (37.2%)	0.507
Diuretics	217 (28.2%)	629 (45.6%)	<0.001

DOAC; Direct Oral Anticoagulant, CCB; Calcium channel blocker, RAAS; Renin-angiotensin system, ACEi; Angiotensin-converting enzyme inhibitor, ARB; Angiotensin II receptor blocker.

**Table 3:** In-hospital outcomes in paroxysmal and non-paroxysmal groups among AF patients.

	Paroxysmal	Non-Paroxysmal
Reason for admission		
Atrial fibrillation	99 (41.9%)	91 (22.9%)
Acute coronary syndrome	39 (16.5%)	54 (13.6%)
Heart failure	13 (5.5%)	74 (18.6%)
Cerebrovascular accident	17 (7.2%)	28 (7.0%)
Systemic embolization other than the brain	0 0. (0%)	5 (1.3%)
Bleeding	2 (0.8%)	18 (4.5%)
COPD	0 (0.0%)	6 (1.5%)
Cardiac operation	0 (0.0%)	2 (0.5%)
Syncope	1 (0.4%)	2 (0.5%)
Non-cardiovascular causes	65 (27.5%)	118 (29.6%)
In Patients outcome		
Discharged home	219 (93.2%)	377 (95.0%)
In hospital death	15 (6.4%)	18 (4.6%)
Cause of in-hospital death		
Acute myocardial infarction	1 (0.4%)	2 (0.5%)
Stroke	3 (1.3%)	6 (1.5%)
Sepsis	9 (3.8%)	4 (1.0%)
Cardiac arrest of an undetermined etiology	1 (0.4%)	1 (0.3%)
Cardiogenic shock	0 (0.0%)	2 (0.5%)
Upper GI bleeding	0 (0.0%)	3 (0.8%)
Acute respiratory failure	1 (0.4%)	0 (0.0%)

COPD, Chronic obstructive pulmonary disease.

**Table 4:** Binary Logistic regression of the predictor factors for having non-paroxysmal atrial fibrillation.

Variable	Odds Ratio	95% CI		*p*-Value
		Lower	Upper	
High risk CHA2DS2-VASc score	1.86	1.39	2.50	<0.001
First AF episode	0.23	0.18	0.30	<0.001
Current smoker	0.97	0.70	1.35	0.874
Left ventricular hypertrophy	1.07	0.84	1.35	0.574
Left atrial size (centimetre)	1.61	1.36	1.92	<0.001
Valvular heart disease	0.83	0.50	1.40	0.502
Palpitations	0.65	0.49	0.87	0.004
Shortness of breath	1.43	1.08	1.89	0.011
Asymptomatic	1.41	1.00	2.01	0.050
Pulmonary Hypertension	1.25	0.94	1.66	0.117
Chronic kidney disease	1.11	0.74	1.66	0.590
Warfarin	2.05	1.55	2.71	<0.001
Beta blockers	1.41	1.06	1.89	0.018
Amiodarone	0.66	0.50	0.87	0.004
CCB	1.60	1.09	2.33	0.015
Digoxin	2.39	1.64	3.49	<0.001
Diuretcs	1.29	1.01	1.66	0.038

CI, Confidence Interval; CCB; Calcium channel blocker.

## Data Availability

Pooled study data, protocol, or statistical analysis plan can be shared upon request at hmakhamreh@hotmail.com and nnalrabadi@just.edu.jo.
